# Supplementary material for: Identification of photoperiod-induced specific miRNAs in the adrenal glands of Sunite sheep (Ovis aries)
Source: Front Vet Sci. 2022 Jul 22;9:888207. doi: 10.3389/fvets.2022.888207 (PMC9354845; doi:10.3389/fvets.2022.888207)
Supplement: Supplementary file 1 [file Data_Sheet_1.ZIP › Supplementary file/miRDeep2 file.zip!/miRDeep2 file/Know_Structure/SP42A3/oar-mir-23b.pdf]

miRBase precursor : oar-mir-23b
Total read count : 17283
oar-miR-23b read count : 17259
remaining reads : 24

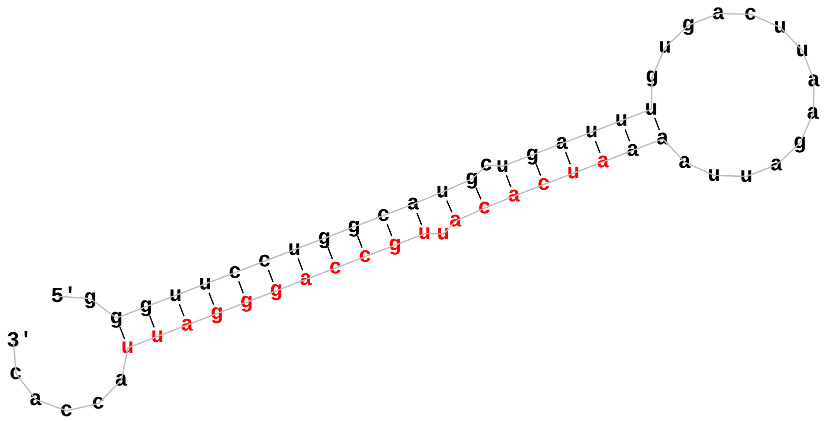

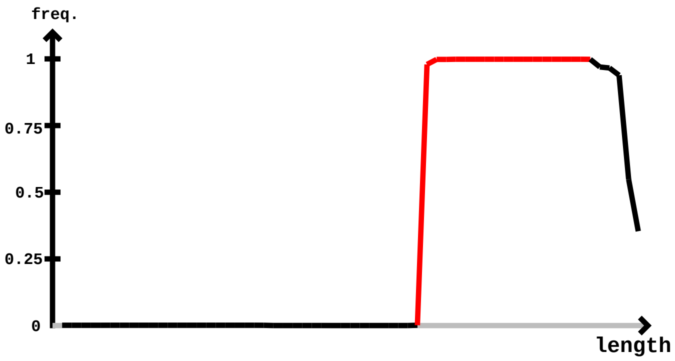


```
                                    oar-miR-23b
5'- ggguuccuggcaugcugauuugugacuuaagauuaaaaucacauugccagggauuaccac   -3'    exp
    ((((((((((((((.((((((..............)))))))).))))))))))..))..    reads      mm         sample
    ggguuccuggcaugcugauu........................................    4          0          seq
    ggguuccuggcaugcugauuu.......................................    15         0          seq
    ggguuccuggcaugcugauuugu.....................................    1          0          seq
    ....uccuggcaugcugauuugugacu.................................    1          0          seq
    ....uccuggcaugcugauuugugacuu................................    1          0          seq
    ...............ugauuugugacuuaagauuaaaa......................    1          0          seq
    ..................................aaaaucacauugccagggauuacc..    1          0          seq
    ....................................aaucacauugccagggauu.....    4          0          seq
    ....................................aaucacauugccagggauuac...    1          0          seq
    ....................................aaucacauugccagggauuacc..    5          0          seq
    ....................................aaucacauugccagggauuacca.    5          0          seq
    ....................................aaucacauugccagggauuaccac    5          0          seq
    .....................................aucacauugccagggau......    5          0          seq
    .....................................aucacauugcUagggauu.....    2          1          seq
    .....................................aucacauugccagggauu.....    489        0          seq
    .....................................aucacauugccUgggauu.....    1          1          seq
    .....................................aucacauugcUagggauua....    1          1          seq
    .....................................aucacauugccagggauua....    62         0          seq
    .....................................aucacauugccagggauuac...    462        0          seq
    .....................................aucacauugccagggauuacc..    6647       0          seq
    .....................................aucacauugccagggauuacca.    3296       0          seq
    .....................................aucacauugccagggauuaccac    5937       0          seq
    ......................................ucacauugccagggauu.....    2          0          seq
    ......................................ucacauugccagggauua....    1          0          seq
    ......................................ucacauugccagggauuac...    7          0          seq
    ......................................ucacauugccagggauuacc..    87         0          seq
    ......................................ucacauugccagggauuacca.    59         0          seq
    ......................................ucacauugccagggauuaccac    169        0          seq
    ........................................acauugccagggauuacc..    7          0          seq
    ........................................acauugccagggauuacca.    1          0          seq
    ........................................acauugccagggauuaccac    4          0          seq
```
